# Enhanced virulence of Autographa californica multiple nucleopolyhedrovirus in *Spodoptera frugiperda* is mediated by an Ac34 mutation that promotes nucleocapsid envelopment within occlusion bodies

**DOI:** 10.1128/jvi.02204-25

**Published:** 2026-05-20

**Authors:** Wenyi Jin, Lin Guo, Yan Tong, Huixian Shi, Uranbileg Ganbold, Qin Kang, Qian Meng, Jihong Zhang, Qilian Qin, Huan Zhang

**Affiliations:** 1 State Key Laboratory of Animal Biodiversity Conservation and Integrated Pest Management, Institute of Zoology, Chinese Academy of Sciences, Beijing, China; 2 University of Chinese Academy of Sciences 74519, Beijing, China; 3 College of Life Sciences, Hebei University, Hebei, China; Wageningen University & Research, Wageningen, Netherlands

**Keywords:** *Spodoptera frugiperda*, AcMNPV, *ac34 *mutation, oral infectivity, virulence evolution, baculovirus biopesticide, nucleocapsid assembly

## Abstract

*Spodoptera frugiperda* poses a significant threat as a polyphagous agricultural pest. Autographa californica multiple nucleopolyhedrovirus (AcMNPV), a broad-spectrum baculovirus with established activity against multiple lepidopteran pests, represents an environmentally benign biocontrol candidate. However, its efficacy against *S. frugiperda* is constrained by limited oral infectivity. Here, serial passaging in *S. frugiperda* yielded a mutant strain of AcMNPV (AcMNPV-Mut) with significantly enhanced oral infectivity relative to the wild strain AcMNPV (AcMNPV-Wt). Importantly, while both oral infectivity and overall virulence (shortened LT₅₀ and increased mortality) against *S. frugiperda* were enhanced, the virus maintained its ability to effectively infect and cause disease in other insect hosts, like *Helicoverpa armigera*. Whole-genome sequencing identified a missense mutation in *ac34*. Functional characterization of a recombinant virus harboring this mutation revealed that the *ac34* variant contributes to virulence augmentation through dual pathways: elevated budded viruses (BVs) production facilitating secondary infection, and upregulated late and very late gene expression, which improves occlusion-derived virus (ODV) embedding within occlusion bodies (OBs), and may lead to an increase in OB size, contributing to a higher viral load per particle, and thereby enhancing primary infectivity. The *ac34* mutation enhances primary and secondary infection, promoting virulence evolution and providing a molecular target for biopesticide design.

**IMPORTANCE:**

In this study, we found that Autographa californica multiple nucleopolyhedrovirus (AcMNPV) undergoes adaptive mutations after serial passages in the semi-permissive host *Spodoptera frugiperda*, driven by mutation in *ac34*. These mutations not only significantly increase viral replication within this pest but also improve the packaging and infectivity of viral particles. This coordinated optimization allows the virus to achieve higher effective doses in a short time, synergistically enhancing its control efficiency against *S. frugiperda*. Importantly, the mutation does not compromise the virus’s pathogenicity toward its original permissive hosts, underscoring its potential utility in single-application strategies for integrated pest management. These findings provide new molecular insights for designing efficient and broadly applicable biocontrol agents, contributing to more sustainable management of agricultural pests and reduced reliance on chemical pesticides.

## INTRODUCTION

Baculoviruses are insect-specific pathogens with a biphasic infection cycle comprising primary and secondary infection phases. During primary infection, larvae ingest occlusion bodies (OBs), which dissolve in the alkaline midgut environment, releasing occlusion-derived virus (ODV), which infects midgut epithelial cells (1). Subsequently, nucleocapsids enter the host cell nucleus, replicate, and produce budded viruses (BVs). These BVs disseminate systemically via the hemolymph to infect various tissues, initiating the secondary infection and resulting in the production of progeny BVs and OBs (2).

The fall armyworm *Spodoptera frugiperda* (Lepidoptera) is a major agricultural pest. Increasing resistance to conventional chemical insecticides has raised serious concerns about environmental safety and pest control efficacy (3). Baculoviruses, such as Autographa californica multiple nucleopolyhedrovirus (AcMNPV), belong to the family *Baculoviridae*, species Alphabaculovirus aucalifornicae, and are promising biocontrol agents due to their high host specificity and environmental friendliness. Notably, although *S. frugiperda* larvae are highly susceptible to BVs-mediated secondary infection, they exhibit reduced susceptibility to oral OBs infections. This suggests that midgut barriers constitute the primary bottleneck for initial infection establishment and horizontal transmission (4). Therefore, selecting fast-acting and highly virulent AcMNPV strains is essential for improving the performance of baculovirus-based biopesticides.

Structurally, baculovirus ODVs are characterized by the presence of either single or multiple nucleocapsids enclosed within a single envelope. In multiple nucleopolyhedroviruses (MNPVs), several nucleocapsids are enclosed within a single envelope, whereas in single nucleopolyhedroviruses, only one nucleocapsid is present per envelope. This structural distinction confers significant infectivity advantages to MNPVs; multi-nucleocapsid ODVs enhance successful infection likelihood through cooperative virion delivery (5). Furthermore, clustered nucleocapsids may facilitate homologous recombination-based DNA repair during replication (6), thereby accelerating genetic diversification and enhancing evolutionary potential.

Previous work (7) has shown that serial passage of AcMNPV-Wt (originally isolated from *Helicoverpa armigera*) in *S. frugiperda* larvae leads to increased virulence, as indicated by reduced median lethal concentration (LC_50_) and shortened median lethal time (LT_50_). This adaptive evolution yielded a mutant strain, AcMNPV-Mut, which significantly enhanced virulence. Whole-genome sequencing revealed three missense mutations (N53D, D54A, T143A) in the *ac34* gene, which are implicated in its enhanced virulence.

In this study, we investigated the molecular mechanisms underlying the increased virulence of AcMNPV-Mut, with a particular focus on the role of *ac34* mutations. We demonstrate that these mutations enhance both BV replication efficiency and the embedding capacity of OB. Specifically, AcMNPV-Mut produces OB containing a significantly higher density of embedded nucleocapsids, which likely facilitates primary infection in the midgut. This structural enhancement may be a key determinant of the increased virulence observed in AcMNPV-Mut. Our findings provide new insights into baculovirus pathogenesis and lay the groundwork for developing more effective baculovirus-based biopesticides for sustainable pest management.

## RESULTS

### AcMNPV adaptive evolution in *S. frugiperda* selects *ac34* mutations that enhance oral infectivity and virulence

**Fig 1:**
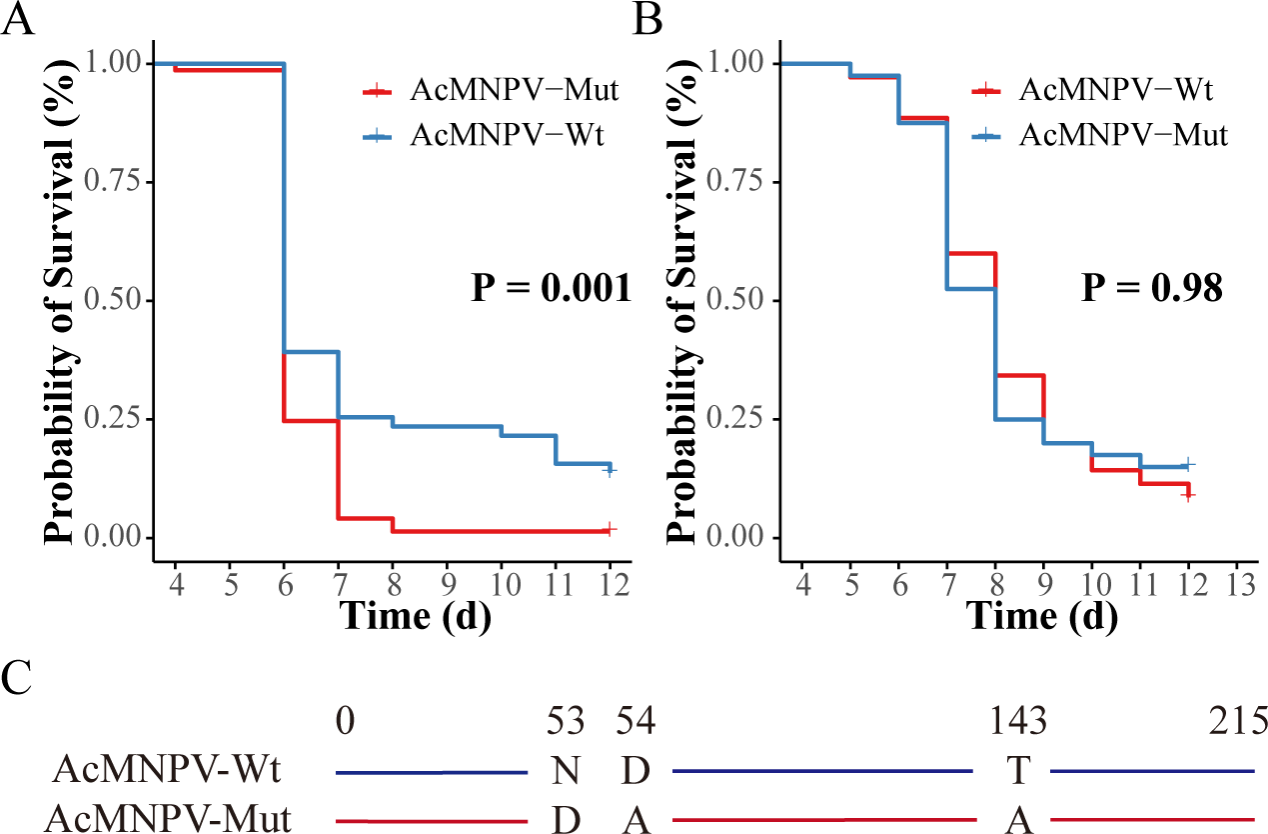
Enhancement of virulence of AcMNPV after serial passaging in *Spodoptera frugiperda*. (**A**) Survival curves of second-instar *S. frugiperda* larvae (*n* = 60) orally infected with OBs of AcMNPV-Wt (propagated in *Helicoverpa armigera*) and AcMNPV-Mut (derived from serial passaging in *S. frugiperda*) at a concentration of 5 × 10⁸ OBs/mL. (**B**) Survival curves of second-instar *H. armigera* larvae (*n* = 60) orally infected with OBs of AcMNPV-Wt and AcMNPV-Mut at a concentration of 1 × 10⁸ OBs/mL. (**C**) Schematic representation of the amino acid substitutions (N53D, D54A, and T143A) identified in the Ac34 protein through comparative whole-genome sequencing of AcMNPV-Wt and AcMNPV-Mut.

Consistent with prior reports on baculovirus evolution in semi-permissive hosts (8, 9), serial passaging (20 generations) of *H. armigera*-derived AcMNPV-Wt through *S. frugiperda* larvae yielded a hypervirulent mutant (AcMNPV-Mut). Notably, our previous work confirmed significantly enhanced oral infectivity of AcMNPV-Mut after serial passage in *S. frugiperda* larvae (7). Both AcMNPV-Mut and AcMNPV-Wt strains were orally administered to late second-instar *S. frugiperda* larvae at a concentration of 5 × 10⁸ OBs/mL. Survival analysis revealed that AcMNPV-Mut caused significantly higher mortality compared to AcMNPV-Wt (Fig. 1A). Furthermore, the LT_50_ analysis revealed that AcMNPV-Mut resulted in a more rapid mortality of larvae, with an LT_50_ of 7.88 ± 0.40 days, in contrast to 9.26 ± 0.28 days for AcMNPV-Wt, and LC₅₀ were 9.21 × 10⁶ and 4.85 × 10⁷ OBs/mL, respectively, further indicating enhanced virulence of AcMNPV-Mut. Inoculation of AcMNPV-Mut into *H. armigera* did not alter its lethality (Fig. 1B), indicating that the adaptive evolution exclusively promoted virulence toward *S. frugiperda* while preserving the inherent broad-spectrum activity of AcMNPV.

To uncover the genetic basis of this increased virulence, whole-genome sequencing was performed on both strains. Comparative genomic analysis of AcMNPV-Mut against the AcMNPV-Wt genome revealed the presence of multiple single-nucleotide polymorphisms in AcMNPV-Mut, the majority of which were synonymous mutations (Fig. 1C). Notably, three missense mutations were identified in the *ac34*, resulting in amino acid substitutions at positions N53D, D54A, and T143A, respectively (Fig. 1C). These findings suggest that *ac34* plays a critical role in the enhanced oral infectivity and pathogenicity of AcMNPV-Mut.

### *ac34* mutation specifically elevates BV titers without affecting OB yield

**Fig 2:**
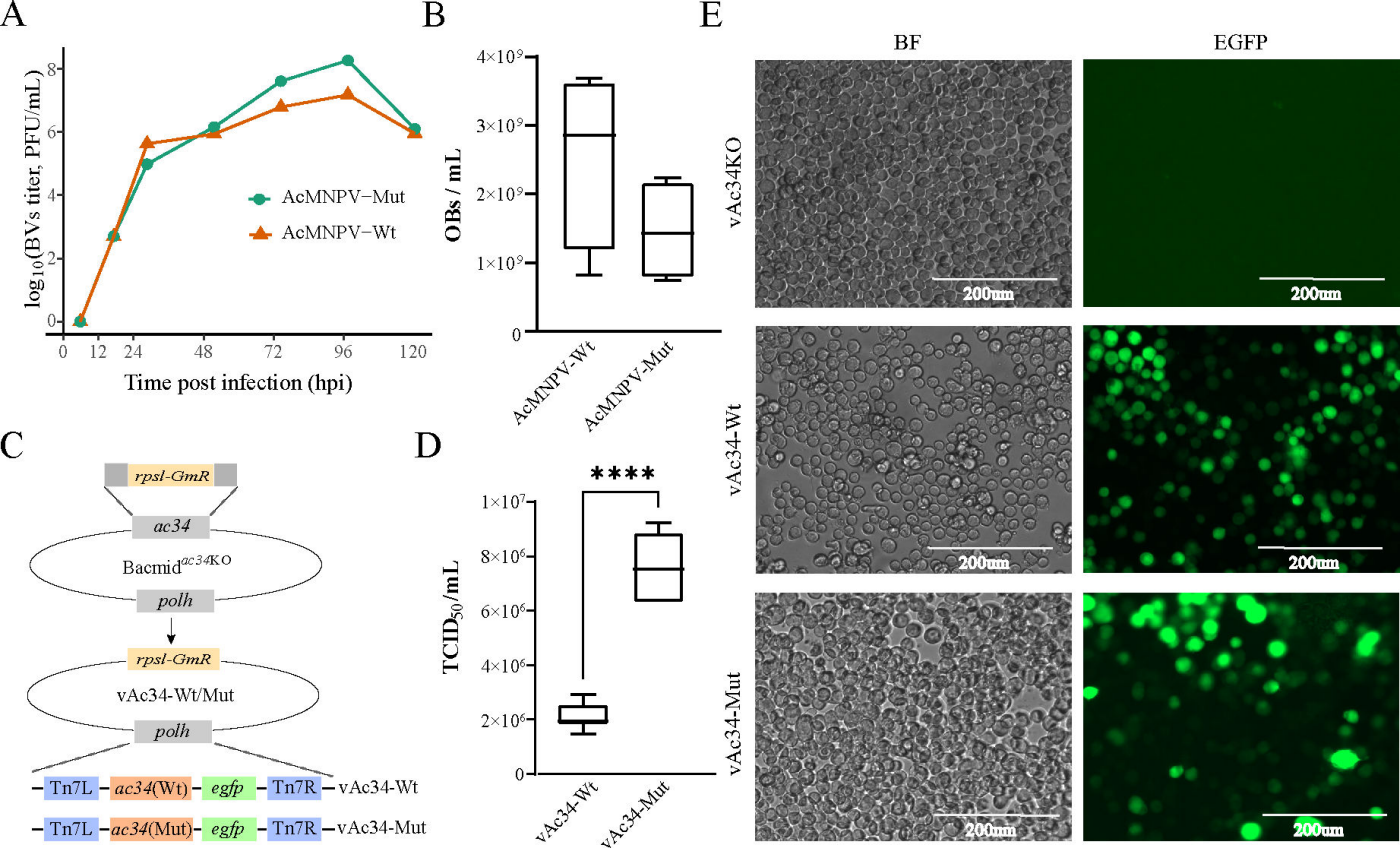
The *ac34* mutation selectively enhances BV titers without affecting OB yield. (**A**) Growth curve of BV titers in Sf9 cells infected with AcMNPV-Wt versus AcMNPV-Mut (MOI = 3). (**B**) Comparison of OBs production in Sf9 cells infected with AcMNPV-Wt versus AcMNPV-Mut (MOI = 3) at 72 hpi. (**C**) Schematic diagram of recombinant bacmid construction strategies: vAc34KO (partial *ac34* deletion), vAc34-Wt (reconstituted with wild-type *ac34*), and vAc34-Mut (reconstituted with mutant *ac34*). (**D**) Comparison of BV titers between vAc34-Wt and vAc34-Mut in Sf9 cells at 72 hpi. (**E**) Fluorescence intensity observations in Sf9 cells transfected with recombinant bacmids (vAc34-Wt and vAc34-Mut), respectively, at 72 hpi. ****, *P* ≤ 0.0001.

To evaluate the impact of serial passaging on AcMNPV replication, equal amounts of BVs from AcMNPV-Wt and AcMNPV-Mut (MOI = 3) were used to infect Sf9 cells. As shown in Fig. 2A, AcMNPV-Mut exhibited significantly higher BV titers at 72 and 96 hpi, reaching 4.0 × 10⁷ BVs/mL, compared to 6.1 × 10⁶ BVs/mL for AcMNPV-Wt (*P* < 0.05). In contrast, the OB yields between the two strains showed no significant difference (*P* > 0.05), with AcMNPV-Mut producing 1.7 × 10⁹ OBs/mL and AcMNPV-Wt yielding 2.17 × 10⁹ OBs/mL (Fig. 2B). This result indicates that the adaptive mutations acquired during serial passaging enhance the replication ability of BVs without affecting OBs formation.

To further investigate the function of *ac34* mutations, recombinant bacmids were generated using ET recombination. A bacmid with partial deletion of *ac34* (vAc34KO) was constructed by replacing *ac34* with Rpsl*-*GmR cassette. Two repair constructs were created: vAc34-Wt and vAc34-Mut, which reintroduced the *ac34* coding sequences from AcMNPV-Wt and AcMNPV-Mut, respectively, into the *polh* locus along with *egfp* reporter gene (Fig. 2C).

Consistent with previous findings (10), vAc34KO-transfected Sf9 cells showed weak green fluorescence even at 72 hpi (Fig. 2E), indicating impaired viral replication due to the loss of *ac34*. In contrast, cells transfected with either vAc34-Wt or vAc34-Mut exhibited strong and comparable fluorescence intensity, suggesting that *ac34* mutations do not affect baculovirus transcription or transfection efficiency (Fig. 2E).

At 72 hpi, the infectious BV titer in vAc34-Mut-transfected cells was approximately 3.7-fold higher than in cells transfected with vAc34-Wt (Fig. 2D). These results confirm that the *ac34* mutations provide a replication advantage to AcMNPV-Mut, directly contributing to its elevated BVs production.

### *ac34* mutation selectively enhances late and very late gene expression

**Fig 3:**
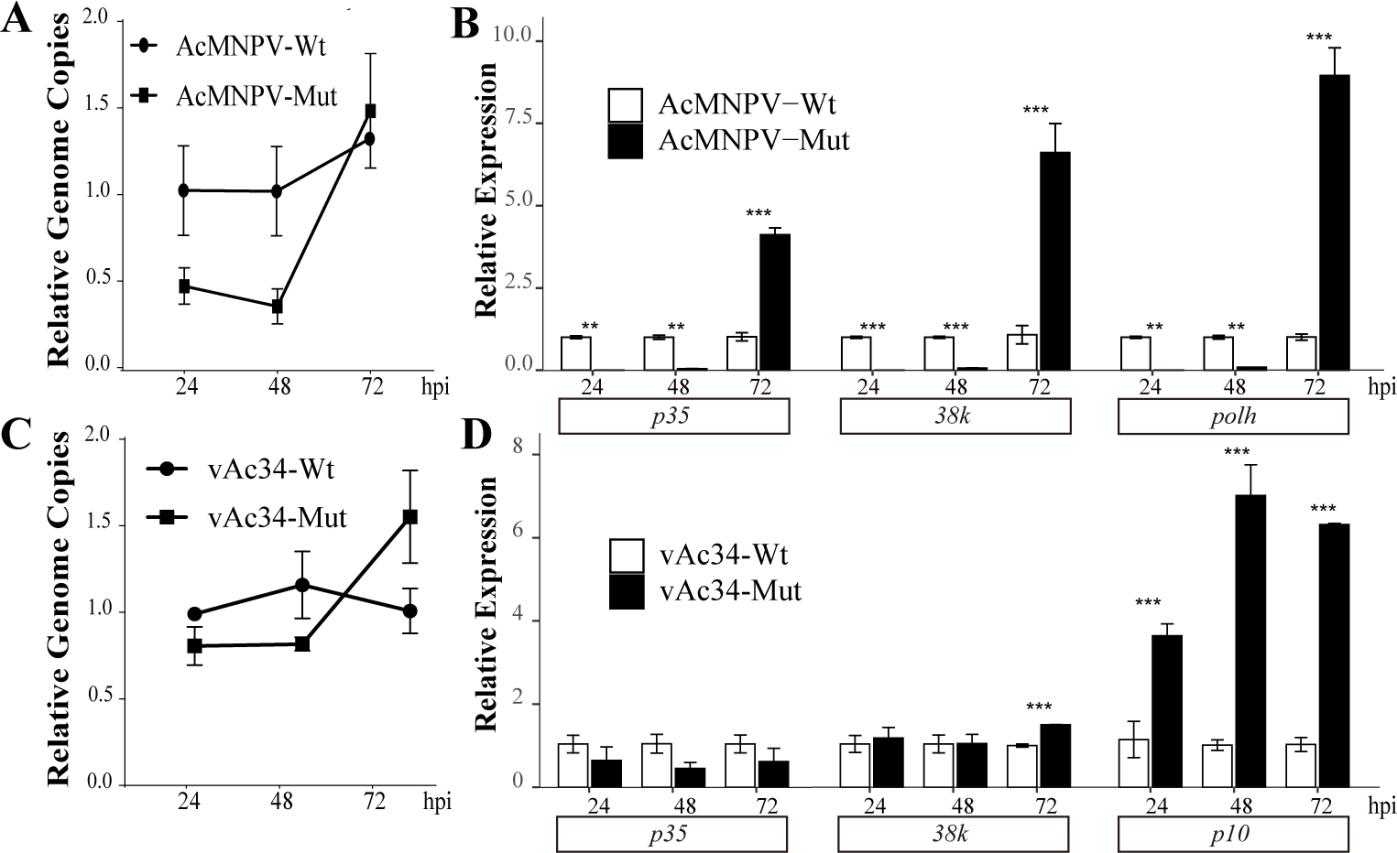
Effects of the *ac34* mutation on viral DNA synthesis and gene expression. (**A**) qPCR quantification of viral genome copy numbers in Sf9 cells infected with AcMNPV-Wt or AcMNPV-Mut (MOI = 3) at 24, 48, and 72 hpi. (**B**) qRT-PCR analysis of early (*p35*), late (*38k*), and very late (*polh*) gene transcripts in Sf9 cells infected with AcMNPV-Wt or AcMNPV-Mut at 24, 48, and 72 hpi. (**C**) qPCR quantification of viral genome copy numbers in Sf9 cells infected with vAc34-Wt or vAc34-Mut (MOI = 3) at 24, 48, and 72 hpi. (**D**) qRT-PCR quantification of early (*p35*), late *(38k*), and very late (*p10*) gene expression in Sf9 cells infected with recombinant viruses vAc34-Wt or vAc34-Mut at 24, 48, and 72 hpi. **, *P* ≤ 0.01; ***, *P* ≤ 0.001.

To evaluate the impact of the *ac34* mutation on viral DNA replication, we quantified viral genome copy numbers in Sf9 cells infected with either AcMNPV-Wt or AcMNPV-Mut. qPCR analysis showed no significant difference in viral genome copies between AcMNPV-Wt and AcMNPV-Mut at any of the time points examined (Fig. 3A). Consistently, recombinant viruses vAc34-Wt and vAc34-Mut yielded comparable results (Fig. 3C), indicating that the *ac34* mutation does not enhance DNA synthesis, suggesting that the observed increase in virulence is independent of DNA replication efficiency.

We next examined whether the *ac34* mutation affects viral transcriptional activity. qPCR was performed to measure transcript levels of representative early (*p35*), late (*38k*), and very late (*polh*) genes in Sf9 cells infected with AcMNPV-Wt or AcMNPV-Mut. All three genes were detectable starting at 24, 48, and 72 hpi in both strains; the transcript levels of all three genes were significantly higher in AcMNPV-Mut than in AcMNPV-Wt (*P* < 0.05, Fig. 3B) at 72 hpi. At this time point, *polh* expression was the highest, followed by *38k* and *p35* in both strains, with a more substantial upregulation of the very late gene in AcMNPV-Mut, suggesting that enhanced gene expression at 72 hpi may contribute to the increased virulence of AcMNPV-Mut. To further elucidate the role of the *ac34* mutations on viral gene expression, recombinant viruses vAc34-Wt and vAc34-Mut were analyzed. qPCR quantification of *p35* and *38k* at 24 and 48 hpi showed no significant differences between the two viruses (*P* > 0.05). In contrast, *38k* and *p10* expression were significantly higher in vAc34-Mut at 72 hpi (*P* < 0.05; Fig. 3D). Collectively, these results demonstrate that the *ac34* mutation selectively enhances the expression of late and, more prominently, very late genes without affecting viral DNA replication. This may be because a substantial portion of the newly replicated naked viral DNA (~70%) is not immediately packaged into progeny virions but instead remains available to support the transcription of genes required for occlusion-related processes (11). This transcriptional bias toward very late genes is likely to boost the production of structural proteins required for OB assembly, thereby facilitating the incorporation of more nucleocapsids into each OB and ultimately increasing oral infectivity.

### Ultrastructural and quantitative analyses reveal that the *ac34* mutation increases OB size and enhances ODV embedding density

**Fig 4:**
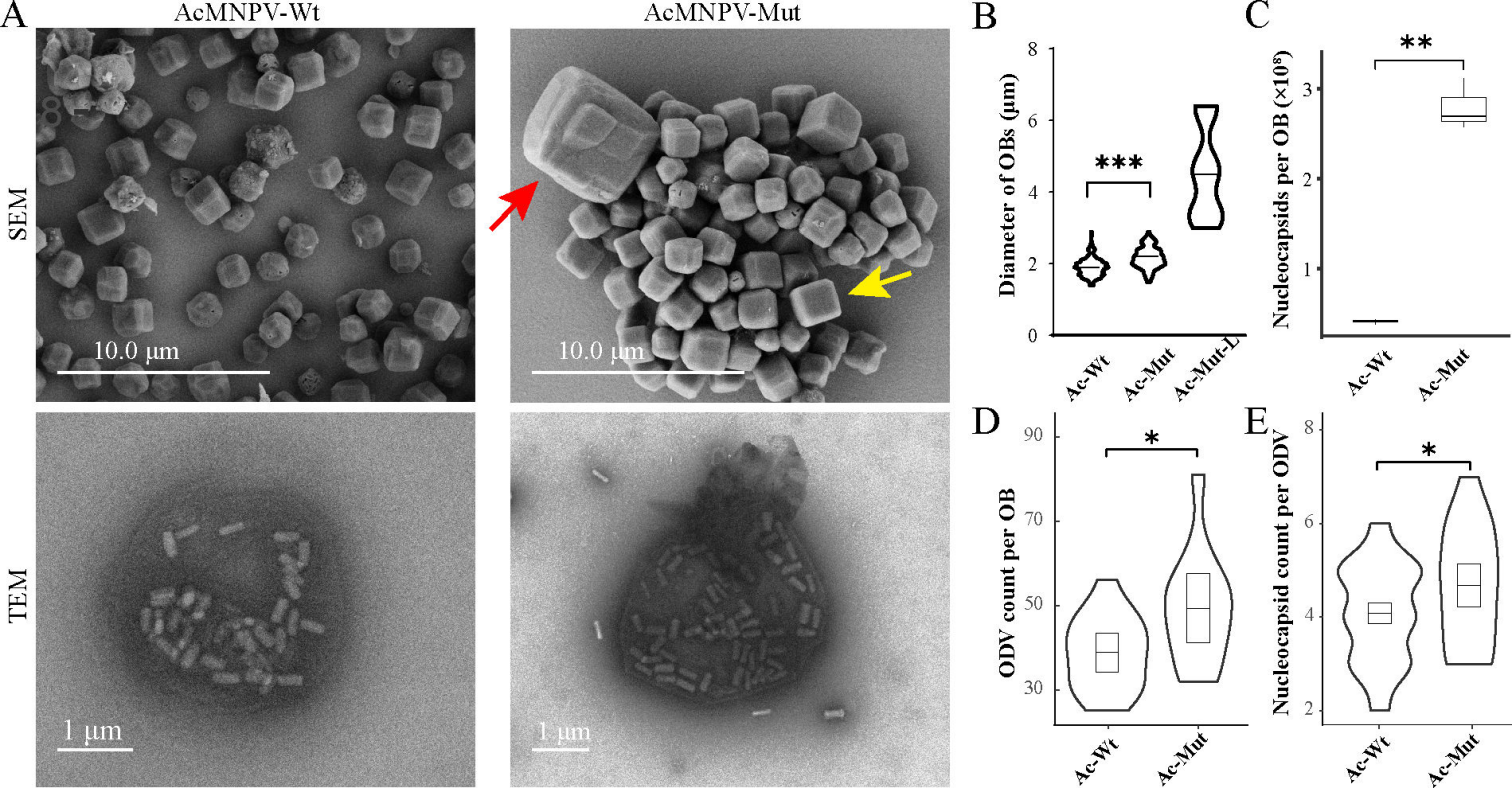
Ultrastructural observation and quantitative analysis of OBs in AcMNPV-Wt and AcMNPV-Mut. (**A**) Representative scanning electron microscopy (SEM, top) and negative-staining transmission electron microscopy (TEM, bottom) images of OBs from AcMNPV-Wt (left) and AcMNPV-Mut (right). Yellow arrows indicate typical AcMNPV-Mut particles, whereas red arrows mark AcMNPV-Mut-L, a subpopulation of unusually large OBs found exclusively in the mutant strain, with diameters approximately twice those of typical AcMNPV-Mut OBs. (**B**) Diameter distribution of OBs from AcMNPV-Wt (Ac-Wt), typical AcMNPV-Mut (Ac-Mut), and enlarged AcMNPV-Mut-L (Ac-Mut-L) particles. (**C**) Quantification of viral genome copy numbers per OB for AcMNPV-Wt (Ac-Wt) and AcMNPV-Mut (Ac-Mut). (**D**) Quantification of the number of ODVs per OB in AcMNPV-Wt (Ac-Wt) and AcMNPV-Mut (Ac-Mut). (**E**) Quantification of nucleocapsid numbers per ODV in AcMNPV-Wt (Ac-Wt) and AcMNPV-Mut (Ac-Mut). *, *P* ≤ 0.05; **, *P* ≤ 0.01; ***, *P* ≤ 0.001.

Scanning electron microscopy (SEM) showed that the OBs of both AcMNPV-Wt and AcMNPV-Mut exhibited the typical polyhedral morphology characteristic of AcMNPV (Fig. 4A and B). Statistical analysis of the particle sizes of 200 OBs from 10 independent fields showed that the average diameter of AcMNPV-Wt OBs was 1.9 ± 0.3 μm, while AcMNPV-Mut OBs were significantly larger, averaging 2.3 ± 0.7 μm (Fig. 4C). The observed difference in size between the two groups was statistically significant. Notably, a subset of abnormally large OBs was observed exclusively in AcMNPV-Mut, with diameters of 4.3 ± 1.2 μm, with a volume approximately 6.5 times that of typical virus particles; this subpopulation was designated AcMNPV-Mut-L (Fig. 4B and C).

Quantitative analysis of viral genome copy number per OB was performed according to Zhang et al. (12) by qPCR. Briefly, equal amounts of OBs (1 × 10^8^) were subjected to alkaline treatment to release the nucleocapsids. Using serial dilutions of pBlunt-fgf plasmid to generate a standard curve (*R*² = 0.999) (Fig. S2), ensuring reliable quantification. Analysis showed that each OB of AcMNPV-Wt contained approximately 1,680 viral genome copies, while that of AcMNPV-Mut reached 12,400 copies (Fig. 4C). These results indicate that the *ac34* mutation enhances ODV embedding density and increases OB size, thereby substantially elevating the viral load per OB.

To further validate the enhanced embedment capacity of the AcMNPV strains after serial passaging, the ODVs liberated from alkali-treated OBs were examined using phosphotungstic acid negative staining. Negative-staining transmission electron microscopy (TEM) analysis showed that an individual AcMNPV-Wt OB contained an average of 30.75 ODVs, compared to 41.31 ODVs for an individual AcMNPV-Mut OB (Fig. 4A and D). Furthermore, the average number of nucleocapsids per ODV was 4.09 for AcMNPV-Wt and 4.68 for AcMNPV-Mut (Fig. 4E).

This negative-staining TEM data aligns with the trend observed in the qPCR-based genome copy number analysis, collectively demonstrating that the *ac34* mutation promotes higher packaging efficiency at multiple structural levels. The observed numerical differences between the absolute genome copies and the physical nucleocapsid counts are likely attributable to the inherent limitations of the qPCR method, which quantifies intact viral genomes, compared to the direct structural count provided by negative-staining TEM.

### *ac34* mutation upregulates envelope-associated genes and enhances ODV embedding efficiency

Nucleocapsid assembly represents a crucial phase in the baculovirus replication cycle, with several genes identified as key regulators for this process. Among them, *ac53* (13), *38k* (*ac98*) (14), and *vp80* (*ac104*) (15) are essential for nucleocapsid formation. Disruption of these genes impairs nucleocapsid assembly and inhibits the production of infectious virions. Additionally, *ac75* (16), *ac76* (17), and *ac93* (18) are involved in the formation of intranuclear microvesicles, facilitating nucleocapsid release and ODV maturation, indicating their important roles in viral maturation. For the membrane encapsulation of nucleocapsids, multiple genes, including *ac11* (19), *ac81* (20), *ody-e25* (*ac94*) (21–23), *ac103* (24), *ac109* (25), and *ac142* (26), are indispensable. Deletion of any of these genes results in defects in ODV membrane formation and prevents proper envelopment into polyhedra. Moreover, the embedding of nucleocapsids into polyhedra is regulated by several key genes, such as *bm133* (27), *bm134* (27, 28), *ac23,* and *ac114* (29) The absence of these genes leads to significant reductions in the number of nucleocapsids enveloped within polyhedra and adversely affects polyhedron morphology. Furthermore, other genes, including *p26* (*ac136*), *p10*, and *p74*, play synergistic roles in ensuring the efficient envelopment of viral particles into polyhedra (30).

**Fig 5:**
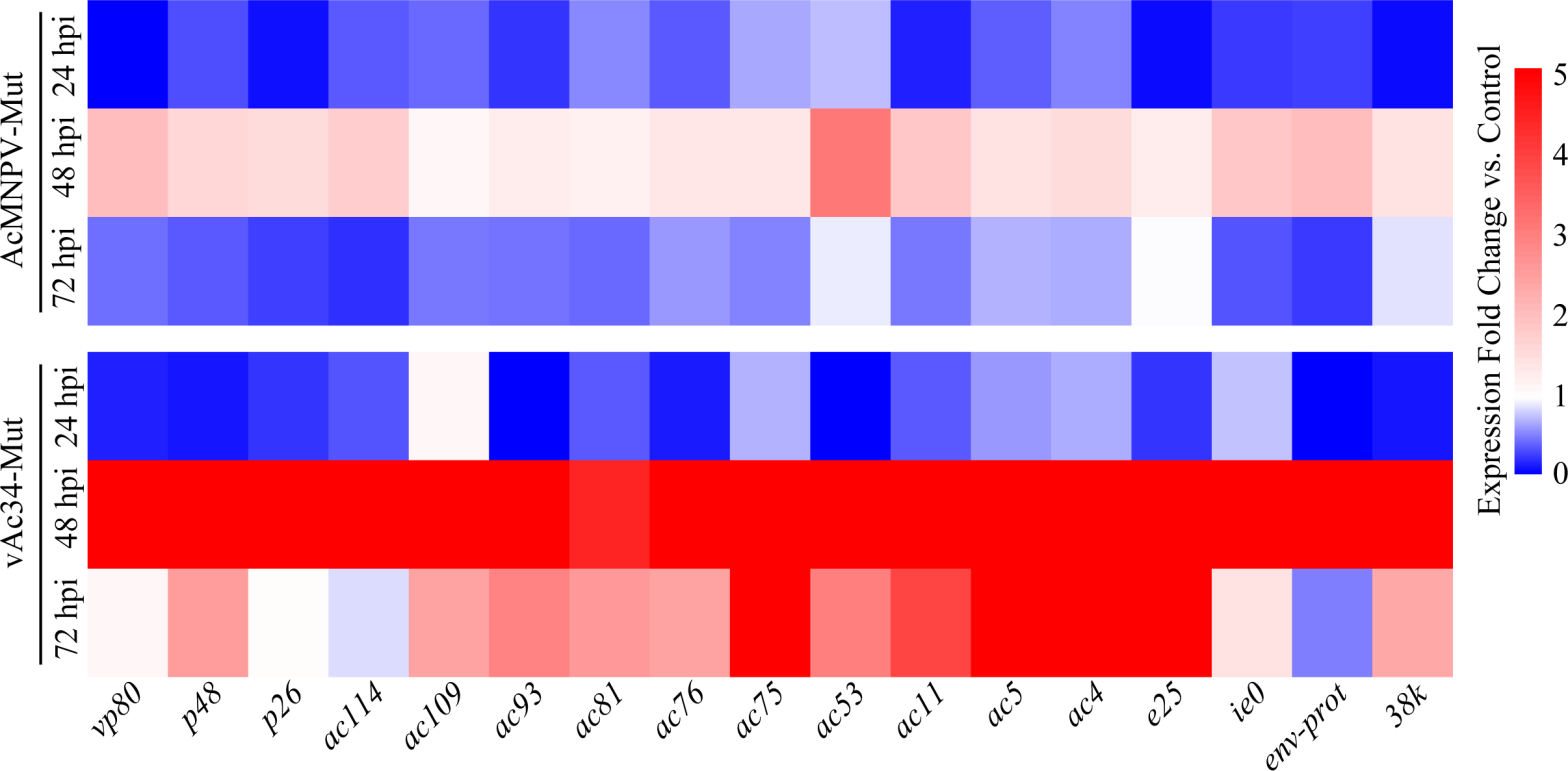
Expression profiles of occlusion-related genes in AcMNPV-Mut and vAc34-Mut. Fold changes in expression of occlusion-related genes in AcMNPV-Mut versus AcMNPV-Wt and vAc34-Mut versus vAc34-Wt at 24, 48, and 72 hpi as determined by qPCR.

To investigate the molecular basis of the enhanced embedment capacity in AcMNPV-Mut, we analyzed the transcript levels of occlusion-related genes at 24, 48, and 72 hpi. Notably, most occlusion-related genes in AcMNPV-Mut were significantly upregulated compared with AcMNPV-Wt at 48 hpi (*P* < 0.05), whereas their expression was lower at 24 and 72 hpi (Fig. 5). This dynamic expression pattern indicates that the regulation of occlusion-related genes in AcMNPV-Mut is time-dependent, exhibiting a marked increase during the mid-infection stage.

To verify whether this time-dependent regulation was attributable to the *ac34* mutation, we examined the same gene set in recombinant viruses vAc34-Wt and vAc34-Mut. As shown in Fig. 5, vAc34-Mut exhibited an expression profile comparable to AcMNPV-Mut, with a marked increase at 48 hpi relative to vAc34-Wt (*P* < 0.05). This time-dependent pattern of gene expression suggests that the *ac34* mutation affects the regulation of occlusion-related genes at specific stages of the viral replication cycle, particularly at 48 hpi, thereby enhancing the virus’s envelopment capacity.

Collectively, these findings demonstrate that the *ac34* mutation enhances viral embedment efficiency by modulating the expression of occlusion-related genes during the late stage of infection. This temporally and spatially regulated enhancement not only improves ODV structural stability and infectivity but also increases the nucleocapsid load per OB, thereby directly elevating the bioavailability of viral particles during oral infection and ultimately driving increased virulence and host adaptation.

## DISCUSSION

In this study, serial passaging of AcMNPV-Wt in semi-permissive host *S. frugiperda* yielded an adapted strain, AcMNPV-Mut, with significantly enhanced oral pathogenicity. Compared with the AcMNPV-Wt, AcMNPV-Mut showed a reduced LT₅₀ by approximately 1.4 days, a 5.2-fold decrease in LC₅₀, and increased mortality rates in *S. frugiperda*. Notably, this enhanced pathogenicity was host-specific, as the intrinsic virulence of AcMNPV in its permissive host *H. armigera* remained unchanged. Such a property provides a practical advantage for pest management, as it enables simultaneous control of co-occurring pests, including *H. armigera*, through a single-application strategy, thereby substantially reducing the complexity of field management. From an applied perspective, the enhanced efficacy of AcMNPV-Mut against *S. frugiperda*, combined with the retained broad-spectrum activity, highlights its potential as a sustainable biocontrol agent. This dual benefit not only lowers reliance on chemical insecticides but also provides a cost-effective, environmentally benign solution for integrated pest management programs, particularly in regions where multiple lepidopteran pests coexist.

Whole-genome sequencing identified three missense mutations in *ac34* (N53D, D54A, T143A) as the key genetic changes in AcMNPV-Mut (7). The *ac34* is highly conserved and essential for nucleocapsid assembly and nuclear localization. The region between residues 91–205 of Ac34 is critical for its nuclear localization, likely due to the presence of a zinc finger motif that may modulate nuclear import signals (31), while deletions in the 206–251 region result in significantly smaller virus plaques (32). Additionally, two synonymous mutation hotspots were identified within the 133–166 region and 545–597 region (32); these hotspots may be associated with functional alterations in Ac34, although the specific role of the 133–166 region remains unclear. Functional dissection of Ac34 in related baculoviruses supports its crucial role: deletion of the bm25 gene, the Bombyx mori homolog of *ac34*, severely compromises viral infectivity (33).

To determine how these mutations enhance virulence, we first examined viral replication. Consistent with previous studies (10, 32), AcMNPV-Mut (and the reconstructed vAc34-Mut) produced substantially higher titers of infectious BVs, suggesting that *ac34* mutations enhance BVs production and thereby facilitate secondary infection and systemic dissemination within the host. This accelerated systemic spread likely contributes to faster host mortality. However, because OB yield was not increased, the elevated virulence cannot be attributed solely to enhanced replication. Indeed, the significantly reduced LC₅₀ indicates that *ac34* mutations also improve the efficiency of primary infection, most likely through enhanced OB quality (5), particularly increased packaging density of nucleocapsids within ODVs and ODVs within OBs.

Transcriptional profiling revealed that AcMNPV-Mut selectively upregulated late and, in particular, very late genes during infection; recombinant vAc34-Mut similarly reproduced this selective enhancement, underscoring the direct role of *ac34* mutations in modulating viral gene expression programs. Notably, a subset of occlusion-related genes was markedly upregulated at 48 hpi, suggesting a time-restricted transcriptional bias that promotes the production of structural and envelope proteins required for nucleocapsid assembly, ODV envelopment, and OB formation (11).

Despite this pronounced upregulation of very late genes, total OB yield was not increased. This apparent discrepancy is likely due to constraints imposed by cellular resources or intranuclear physical space, which limit OB number. Instead of increasing OB quantity, the additional structural proteins produced in AcMNPV-Mut infected cells appear to be preferentially allocated to enlarging individual OBs and enhancing ODV and nucleocapsid packing density. This strategy, which favors increased per-OB infectious capacity rather than OB number, substantially increases the infectious dose delivered by each OB, thereby enhancing oral infectivity without altering total OB production.

Ultrastructural and quantitative analyses corroborated these molecular findings. AcMNPV-Mut produced significantly larger OBs and contained 6.9-fold more genome copies per OB. Moreover, AcMNPV-Mut OBs packaged more ODVs per OB and more nucleocapsids per ODV. These multi-layered enhancements collectively explain the dramatic increase in viral genome copies per OB and demonstrate that the *ac34* mutations fundamentally improve the efficiency of virion assembly and occlusion.

Strikingly, we identified a rare subpopulation of unusually large OBs (Mut-L) corresponding to a 6.5-fold increase in volume. Although these oversized OBs constitute only a very small fraction of the total OB population, we interpret Mut-L as an extreme morphological manifestation of the enhanced packaging efficiency conferred by *ac34* mutations. While infrequent, such a rare subpopulation of oversized OBs (Mut-L) particles may play a disproportionate role during natural oral infection, particularly when hosts ingest very low numbers of OBs. In this context, Mut-L particles could deliver exceptionally high virion doses to midgut epithelial cells, thereby markedly increasing the probability of successful primary infection in a semi-permissive host such as *S. frugiperda*.

The functional consequence of this packaging superiority is a substantially increased probability of successful primary infection per OB. Since OBs serve as the environmental transmission units that initiate infection in the midgut, a higher viral load per OB can directly enhance oral infectivity (5).

Together, these data support a model in which *ac34* mutations reprogram late-stage viral transcription to favor nucleocapsid production, ODV envelopment, and efficient OB assembly. One plausible mechanism involves altered interactions with host or viral regulators of nuclear organization and actin dynamics, such as CRM1-mediated nuclear export and Arp2/3-dependent nuclear actin polymerization (34–36), which have been implicated in nucleocapsid trafficking and intranuclear assembly. Modified Ac34 function may shift the timing or amplitude of these processes, concentrating assembly machinery during a critical mid-to-late infection window and thereby increasing envelopment efficiency.

In conclusion, *ac34* mutations drive adaptive virulence evolution by synchronously enhancing BV production and increasing the packaging efficiency of ODVs and nucleocapsids into OBs. This dual enhancement of secondary spread and primary infection efficacy provides a mechanistic explanation for the rapid adaptation of AcMNPV to *S. frugiperda* under serial passaging selection, while preserving its intrinsic broad-spectrum activity. Such a combination of host-adapted virulence and conserved generalist infectivity underscores the potential of AcMNPV-Mut as a practical and sustainable biocontrol agent, offering improved field efficacy and reduced management complexity in cropping systems challenged by multiple lepidopteran pests.

## MATERIALS AND METHODS

### Bacterial strains, bacmid DNA, and primer sequences

*Escherichia coli* DH5α and TOP10 (TransGen, China) were used in all cloning procedures. *E. coli* BW25113 was kindly provided by Prof. Manli Wang, Wuhan Institute of Virology, Chinese Academy of Sciences, Wuhan, China. High-fidelity DNA polymerases, restriction endonucleases, and DNA-modifying enzymes were purchased from New England Biolabs (USA). Primer sequences are listed in Table S1.

### Viruses, insect cells, and transfection

The *S. frugiperda* IPLBSf21-AE clonal isolate 9 (Sf9) cell line was cultured at 27°C in Insect-XPRESS medium (12-730Q, BioWhittaker, Lonza, USA) supplemented with 10% fetal bovine serum, 100 U/mL penicillin, and 100 µg/mL streptomycin. Wild-type AcMNPV (AcMNPV-Wt) and its mutant strain (AcMNPV-Mut) were generated and maintained in our laboratory. AcMNPV-Wt was derived from a monoclonal isolate obtained from *H. armigera*. Larval cadavers exhibiting typical baculovirus-induced symptoms (melanization and liquefaction) were collected and used for 20 serial passages in third-instar *S. frugiperda* larvae to obtain AcMNPV-Mut. Following these passages, BVs were harvested, cloned, and injected into *S. frugiperda* larvae, and the resulting virus was designated AcMNPV-Mut as described previously (7). *H. armigera* and *S. frugiperda* larvae were reared on an artificial diet at 25°C.

### Bioassays

Overnight-starved second-instar larvae at the pre-molt stage were collected. Newly molted third-instar larvae were fed an artificial diet containing 5 × 10⁸ OBs/mL of either AcMNPV-Wt or AcMNPV-Mut. Control larvae received a virus-free diet. Ninety larvae per treatment group were maintained on a virus-supplemented diet for 24 h, followed by transfer to a normal diet. Larval mortality was recorded, and survival curves were generated using the Kaplan-Meier method.

### Virus proliferation curve

Sf9 cells were infected with AcMNPV-Wt, AcMNPV-Mut, or recombinant viruses (vAc-Wt and vAc-Mut) at an MOI of 3. After 2 hpi, cells were washed and cultured further. At 12, 24, 48, 72, 96, and 120 hpi, supernatants containing BVs were collected by centrifugation, and pellets containing OBs were retained. BV titers were determined by plaque assay (37). OBs were released from cell pellets by 1% SDS lysis and counted with a hemocytometer.

### Construction of recombinant viruses

The ac34(U)-rpsl-GmR-ac34(D) fragment was amplified from the p-Rpsl-GmR plasmid using primers Rpsl-GmR-F/R. This fragment, containing *ac34* flanking sequences, was used to replace the *ac34* gene (nt 28448–28692) in AcMNPV via RecE/RecT recombination (35), generating the knockout bacmid bAc34KO. Kanamycin and gentamicin selection was applied. bAc34KO was transformed into TOP10 cells carrying the helper plasmid pMON7124, and transformants were selected with gentamicin and tetracycline to yield competent DH10Bac (Ac34KO).

Wild-type and mutant *ac34* fragments were amplified from AcMNPV-Wt and AcMNPV-Mut genomic DNA, respectively, using primers Ac34-F/R. The *egfp* fragment was amplified with primers EGFP-F/R. Fusion fragments [Ac34 (Wt)-EGFP and Ac34 (Mut)-EGFP] were generated by bridging PCR, digested with KpnI and BamHI, and ligated into pFB-EGFP. Ligated products were transformed into DH5α, and ampicillin-resistant colonies were used to prepare donor plasmids. These donor plasmids were transposed into the *polh* locus of bAc34KO to produce bAc34KO-Ac34 (Wt)-EGFP and bAc34KO-Ac34 (Mut)-EGFP, which were transfected into Sf9 cells using Cellfectin II (Invitrogen, USA). Recombinant viruses vAc34KO, vAc34-Wt, and vAc34-Mut were harvested from supernatants at 96 h post-transfection.

### Quantification of viral DNA replication

Sf9 cells were infected with AcMNPV-Wt or AcMNPV-Mut at an MOI of 3. At 24, 48, and 72 hpi, cell pellets were collected, lysed in 1% SDS, and OBs were recovered by centrifugation. DNA was extracted via phenol-chloroform following alkaline lysis (NaOH, pH 10.8, 37°C, 1 h). qPCR was performed using primers FGF-F/R, with *rpl3* as the internal control, following the UltraSYBR Mixture protocol (Takara, Japan).

### RNA extraction and reverse transcription-PCR

Sf9 cells were infected with AcMNPV-Wt, AcMNPV-Mut, vAc34-Wt, or vAc34-Mut at an MOI of 3. At 24, 48, and 72 hpi, total RNA was extracted with TRIzol reagent, and cDNA was synthesized using the SuperRT cDNA Synthesis Kit (Kangwei, China). Transcript levels were determined by qPCR, normalized to *rpl3* expression. Primers are listed in Table S1.

### Electron microscopy

Purified OBs were examined by light microscopy (Leica DM2000, Leica, Germany) for preliminary assessment. For scanning electron microscopy (SEM; Hitachi, Japan), OBs were imaged at 5 kV. OBs dimensions were measured from 200 particles across 10 randomly selected fields.

For negative-staining transmission electron microscopy (TEM, ThermoFisher, USA), OBs were dropped onto carbon-coated copper grids and incubated for 3 min. Samples were then treated with 0.5× DAS lysis buffer (0.1 M Na₂CO₃, 0.01 M EDTA, 0.15 M NaCl, pH 11) at 37°C for 20 min. After 2 min of staining by 3% phosphotungstic acid negative stain, grids were examined at 120 kV.

Statistical analysis was conducted using GraphPad Prism 8.0.2; differences between groups were assessed by ANOVA and Student’s *t*-test, with *P* < 0.05 considered significant.

### Quantification of nucleocapsids

Nucleocapsids per unit OB were quantified by qPCR as described previously (12). A standard curve was generated using serial dilutions of plasmid pBlunt-fgf, constructed by cloning the *fgf* fragment into pEASY-Blunt (TransGen, China). OBs from AcMNPV-Wt and AcMNPV-Mut were digested by alkaline hydrolysis, and nucleocapsid copy numbers were determined by qPCR.

## Data Availability

No new genome sequences were generated in this study. Information on the mutations in *ac34* is provided in Fig. S1. Other data are available from the corresponding author upon reasonable request.

## SUPPLEMENTAL MATERIAL

Fig. S1 jvi.02204-25-s0001.tif Single-nucleotide substitutions identified in *ac34* at the nucleotide level.

Fig. S2 jvi.02204-25-s0002.pdf Standard curve.

Table S1 jvi.02204-25-s0003.xlsx Primer sequences.

ASM does not own the copyrights to Supplemental Material that may be linked to, or accessed through, an article. The authors have granted ASM a non-exclusive, world-wide license to publish the Supplemental Material files. Please contact the corresponding author directly for reuse.
