# Supplementary figures and images for: Enhanced virulence of Autographa californica multiple nucleopolyhedrovirus in Spodoptera frugiperda is mediated by an Ac34 mutation that promotes nucleocapsid envelopment within occlusion bodies
Source: J Virol. 2026 May 20;100(6):e02204-25. doi: 10.1128/jvi.02204-25 (PMC13289059; doi:10.1128/jvi.02204-25)

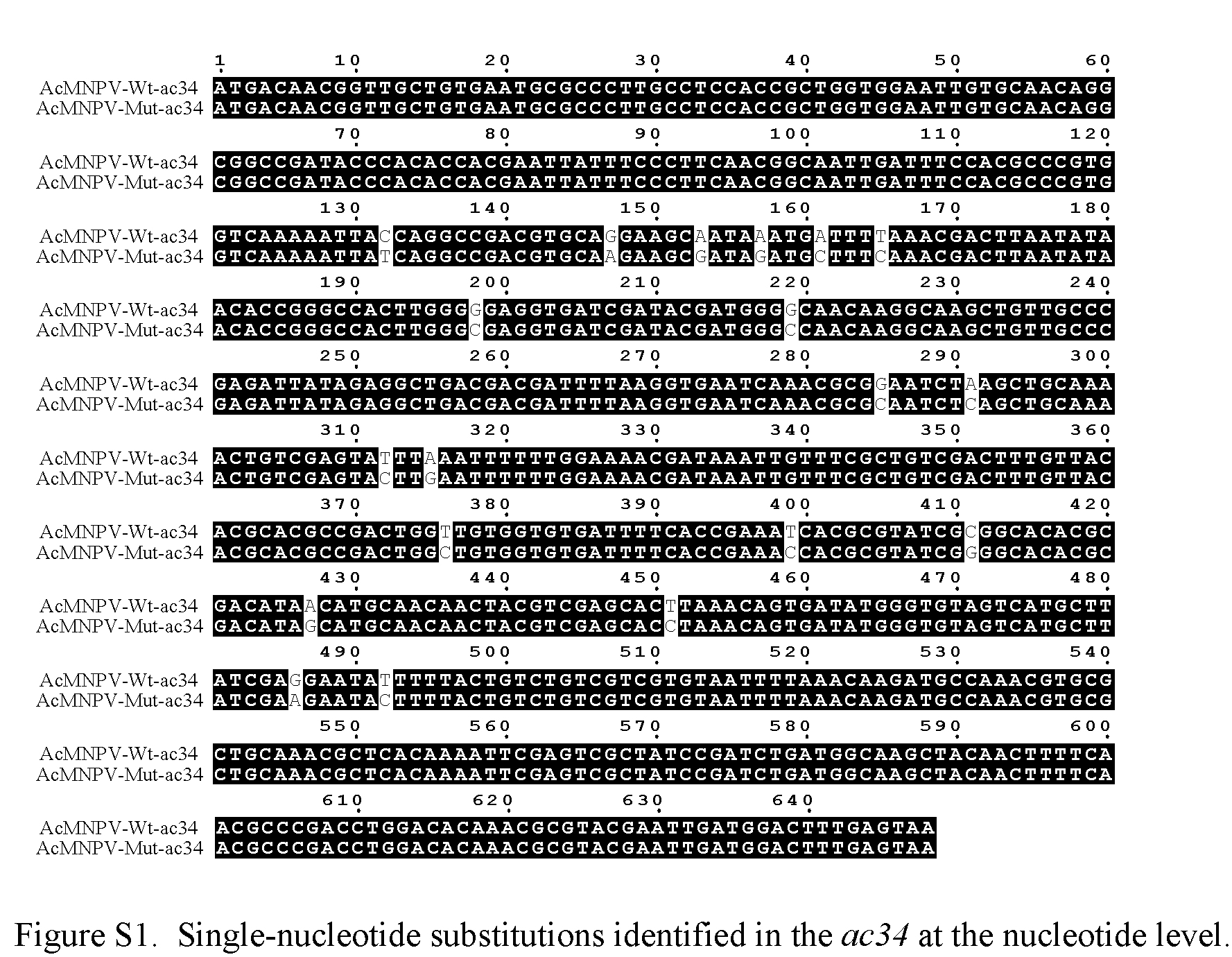

Supplement: Fig. S1 — Single-nucleotide substitutions identified in ac34 at the nucleotide level. [file jvi.02204-25-s0001.tif]
