# Supplementary material for: Enhanced virulence of Autographa californica multiple nucleopolyhedrovirus in Spodoptera frugiperda is mediated by an Ac34 mutation that promotes nucleocapsid envelopment within occlusion bodies
Source: J Virol. 2026 May 20;100(6):e02204-25. doi: 10.1128/jvi.02204-25 (PMC13289059; doi:10.1128/jvi.02204-25)
Supplement: Fig. S2 — Standard curve. [file jvi.02204-25-s0002.pdf]

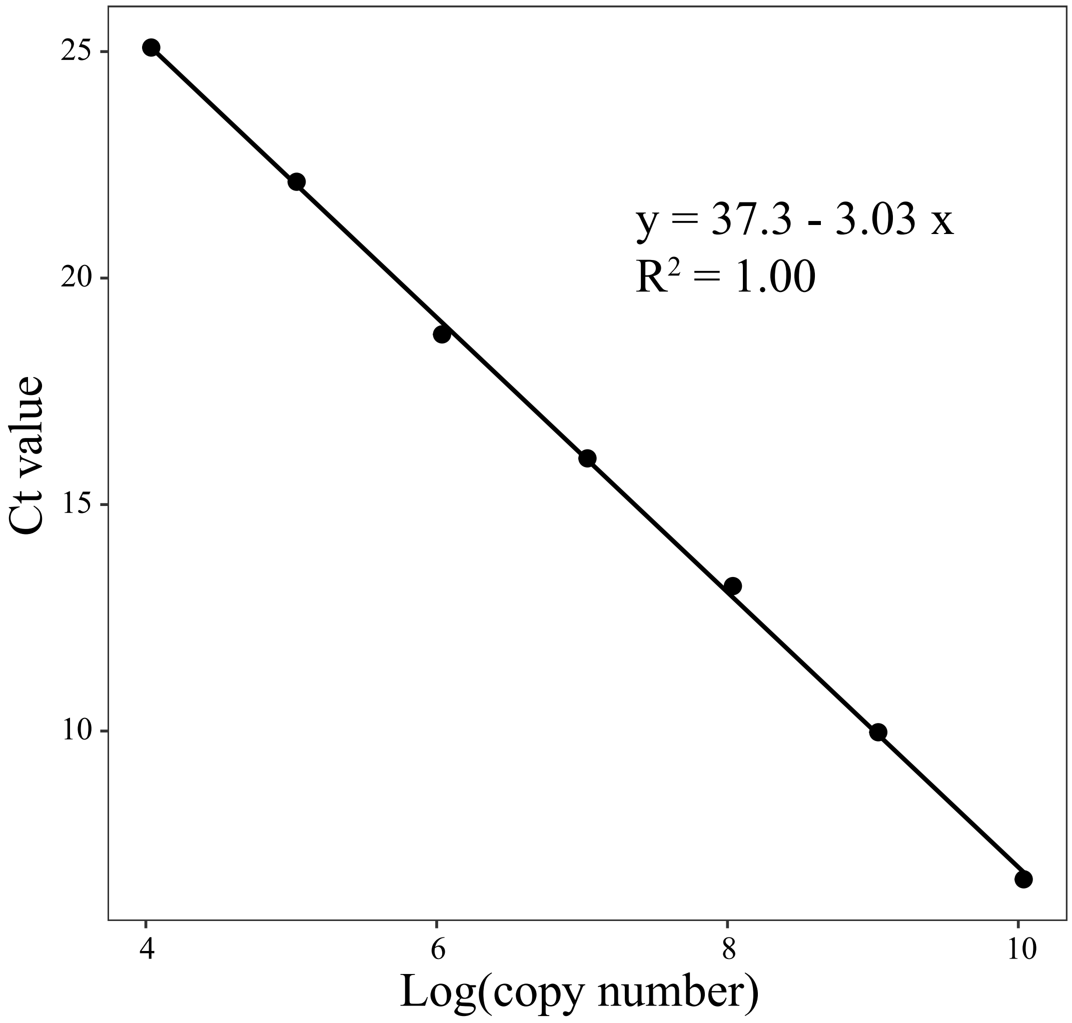

Figure S2. The standard curve was generated by plotting Ct values against the template copy number.The limit of detection of the assay was 10^3 copies.
